# Controlling Multi-Drug-Resistant Traits of *Salmonella* Obtained from Retail Poultry Shops Using Metal–Organic Framework (MOF) as a Novel Technique

**DOI:** 10.3390/microorganisms11102506

**Published:** 2023-10-07

**Authors:** W. Kamal, Rehab Mahmoud, Abeer Enaiet Allah, Ahmed A. Farghali, Abdalla Abdelwahab, Dalal Hussien M. Alkhalifah, Wael N. Hozzein, Manar Bahaa El Din Mohamed, Sahar Abdel Aleem Abdel Aziz

**Affiliations:** 1 Department of Chemistry, Faculty of Science, Beni-Suef University, Beni-Suef 62514, Egypt; wesamkamal16@yahoo.com (W.K.); abeer.abdelaal@science.bsu.edu.eg (A.E.A.); 2 Materials Science and Nanotechnology Department, Faculty of Postgraduate Studies for Advanced Science (PSAS), Beni-Suef University, Beni-Suef 62511, Egypt; ahmedfarghali74@yahoo.com (A.A.F.); abdalla.abdelwahab85@gmail.com (A.A.); 3 Faculty of Science, Galala University, Sokhna 43511, Egypt; 4 Department of Biology, College of Science, Princess Nourah Bint Abdulrahman University, P.O. Box 84428, Riyadh 11671, Saudi Arabia; dhalkalifah@pnu.edu.sa; 5 Department of Botany and Microbiology, Faculty of Science, Beni-Suef University, Beni-Suef 62511, Egypt; hozzein29@yahoo.com; 6 Department of Hygiene, Zoonoses and Epidemiology, Faculty of Veterinary Medicine, Beni-Suef University, Beni-Suef 62511, Egypt; dr.manarbahaa@gmail.com (M.B.E.D.M.); abdelaziz.sahar@yahoo.com (S.A.A.A.A.)

**Keywords:** antimicrobial, resistance, *Salmonella*, retail poultry shop, biofilm, MOF

## Abstract

*Salmonella* spp. is considered one of the most important causes of food-borne illness globally. Poultry and its products are usually incriminated in its spread. Treatment with antibiotics is the first choice to deal with such cases; however, multi-drug resistance and biofilm formation have been recorded in animals and humans. This study aimed to detect the antibiotic profile of isolated traits from different sources and to find innovative alternatives, such as MOFs. A total of 350 samples were collected from randomly selected retailed poultry shops in Beni-Suef Province, Egypt. Their antimicrobial susceptibility against eight different antibiotics was tested, and multi-drug resistance was found in most of them. Surprisingly, promising results toward MOF were detected. Cu/Ni/Co-MOF (MOF3) showed superior antibacterial efficiency to Cu/Ni-MOF (MOF2) and Cu-MOF (MOF1) at *p* value ≤ 0.01. These findings highlight the tendency of *Salmonella* spp. to develop MDR to most of the antibiotics used in the field and the need to find new alternatives to overcome it, as well as confirming the ability of the environment to act as a source of human and animal affection.

## 1. Introduction

Many pathogenic infections with the rapid and escalating development in have threatened the poultry industry over time, including viral, bacterial, parasitic, and fungal infections [1]. *Salmonellosis* is mainly connected to poultry production. It is associated with a high mortality rate that can reach up to 90.0%, causing severe economic losses to the producers [2,3]. *Salmonella* species are one of the most important food-borne pathogens in the entire world and one of the main causes of infectious gastroenteritis in humans, largely due to the human consumption of contaminated poultry products such as eggs and raw or insufficiently cooked chicken [4,5]. Noticeably, *salmonellosis* in humans is mainly associated with the consumption of poultry products contaminated with *S. Typhimurium* and *S. enteritides* and is considered a major public health concern [2,3]. Antimicrobial resistance is a worldwide concern for both humans and animals, especially with the increasing risk of transfer of the genes responsible for this resistance among different bacterial genera and subsequent failure of treatments and increase in the costs of health care, as well as animal deaths and economic losses [6,7]. Some reports have predicted that deaths due to antimicrobial-resistant bacteria will reach up to 10 million in the year 2050 [8]. Several synthesized substances have been used to fight microbes and bacteria (e.g., *Shigella*) [9,10].

Therefore, there is a need to find alternatives to antibiotics that are ineffective against bacteria, particularly *Salmonella* spp. One of the best materials to have emerged in the field of biomedicine is metal–organic frameworks (MOFs). They have a long-term antibacterial and biocompatible potential due to their unique morphology and specific physical and chemical properties that can kill bacteria efficiently via physical interaction and the slow release of metal ions [11,12]. MOFs have a porous low-density crystalline structure made of bipedal or multimodal organic ligands as organic linkers between the metal nodes [13,14,15]. MOFs have been thoroughly studied and used in a variety of fields, including gas storage, adsorption and separation, sensing, organo-catalysis, photocatalysis, contaminant removal from water or gas, and the field of electrochemistry, because of their abundant coordination-unsaturated metal sites, tunable porous structure, high specific surface area, and tunable substitutable components [16,17,18]. Because of their superior biodegradability, low toxicity, and ease of functionalization, their uses have expanded to the fields of medicine and biology [19,20]. Moreover, the controlled/stimulated decomposition, strong interaction with bacterial membranes, ROS production under irradiation, as well as high loading amounts and controlled release of other antibacterial agents render MOF an ideal material for various antibacterial applications in the field of biomedicine [11,20]. Additionally, MOFs can act as efficient carriers for the controlled delivery of active substances associated with their high drug loadings compared to alternative materials [21,22]. The general mechanism by which bioactive MOFs work is the release of bioactive metal ions or ligands into the media after the breaking of metal–ligand linkages [23,24]. Numerous antibacterial composite MOF materials have been investigated by mixing a range of chemicals and antibacterial components, including nanoparticles [25], antibiotics [26], phytochemicals [27], and polymers [28], with Ag-MOFs-polyetherimide (PEI) [29] as an example. Metal ions and ligands have been identified as the main elements that significantly affect the sterilization ability of MOFs for bacterial inactivation. In recent years, the antibacterial mechanism of MOFs has also been proposed. Excellent physical–chemical characteristics of MOF materials allow them the overcoming of the drawback of antibiotic drug resistance [26] and producing potent antibacterial activity. They primarily rely on van der Waals forces and electrostatic and hydrophobic interactions between MOFs and bacteria [30,31,32]. Additionally, MOFs have a long-lasting antibacterial property and can be used as a metal storage container for the slow release of Ag, Co, Cu, and Ni ions [33]. The released metal ions can slowly penetrate the cell membrane and destroy cell components. Different metal ions exhibit different mechanisms of activation. As an example, Cu^2+^ ions dispersed in an aqueous solution might have the ability to generate ROS to interfere with the synthesis of DNA and amino acids [34]. The Co^2+^ ions interact with the PO_4_^3−^ of the phospholipids of the bacterial membrane to cause pro-oxidative stress and reactive oxygen species (ROS) [35]. Recent research has focused on additional metals, such as Cu and Ni, that might have antibacterial properties [36]. Therefore, in this study, we focused on pure MOF (Cu-MOFs), bimetallic MOF (Cu/Ni-MOFs), and ternary metallic MOF (Cu/Ni/Co-MOFs). In bimetallic metal–organic frameworks, two distinct metal ions are present in the inorganic nodes of Cu/Ni-MOF. In order to change the coordination environments and electrical properties of the active sites and further enhance activity, third metal ions were purposely introduced to the MOF known as the trimetallic MOFs (Cu/Ni/Co-MOF matrix) [37].

Therefore, this study aimed to determine the antibiotic profile of isolated traits from different animal, human and environmental samples and to find a novel alternative to ineffective ones through synthesis, characterization of Cu/MOFs, Bimetallic Cu/Ni-MOFs and Ternary metallic MOF Cu/Ni/Co/MOFs and to study the biocidal effect those MOFs against MDR *Salmonella* spp. 

## 2. Materials and Methods

### 2.1. Materials

Loba chemie (Mumbai, India) was the source of Cu (NO_3_)_2_·4H_2_O, Alpha chemical (Cairo, Egypt) for Ni(NO_3_)_2_·6H_2_O and Co(NO_3_)_2_·6H_2_O from Pio chem (6th of October City, Egypt). DMF (dimethylformamide) was purchased by Carlo Erba (Emmendingen, Germany). 2-aminoterphthalic acid, an organic ligand, was bought from Sigma Aldrich (Cairo, Egypt). NaOH was supplied by Merck (Darmstadt, Germany). Distilled water was used to make all preparations. Media used for isolation, identification of *Salmonella* as well as antibiotic discs buffered peptone water (BPW) and Xylose–lysine–deoxycholate (XLD) agar plates were purchased from Oxoid LTD (Basingstoke, UK). The DNA extraction kits was obtained from Qiagen, (Hilden, Germany, GmbH).

### 2.2. Study Area and Period

This study was carried out in Beni-Suef city (coordinates 29°04′ N–31°05′ E), Beni-Suef province, Egypt, during the period from April to December 2022.

### 2.3. Sample Collection

Samples were collected from randomly selected retail poultry shops in Beni-Suef City, Egypt. In total, 350 samples were gathered comprising intestinal swabs (*n* = 140), environmental samples (*n* = 160) including swabs obtained from cutting knives (*n* = 40), cutting board (*n* = 40), rinsing water (*n* = 40) and defeathering machine (*n* = 40). Regarding human samples, 50 hand swabs were collected from poultry shop workers with oral consent. Samples were individually collected in plastic screw-cupped tubes containing sterile 10 mL of buffered peptone water (BPW) (Oxoid Ltd., Basingstoke, UK). The samples were directly transported to the Animal Hygiene and Zoonoses Laboratory, Faculty of Veterinary Medicine, Beni-Suef University, to isolate *Salmonella*. Data were gathered from the shop workers, including their age, gender, location of residence, and the recorded signs and symptoms. The standards of hygienic measures that prevailed in the shops under study were recorded, and to a large extent, they were similar, ranging from very low to low. None of the examined workers had an apparent illness.

The study protocol was approved by the International Animal Care and Use Committee (IACUC). No. 022-411 and the Institutional Review Board (IRB) of Beni-Suef University.

### 2.4. Isolation, Identification, and Serotyping of Salmonella Isolates

In total, 0.1 mL of the inoculated BPW broth was transferred into a tube containing 10 mL of the Rappaport Vassiliadis broth (RVB) medium and all tubes were incubated at 41.5 °C for 24 h. Then, Xylose–lysine–deoxycholate (XLD) agar plates (Oxoid Ltd., Basingstoke, UK) were streaked with a loopful of RVB and left to incubate for 24 h at 37 °C. The morphological and cultural characteristics of the colonies were investigated. Gram staining and biochemical identification were used to identify the red colonies with black centers that were thought to be *Salmonella* colonies [38]. Furthermore, slide agglutination with standard “O” and “H” antisera was used to serotype the suspected *Salmonella* isolates using the Kauffman–Whitescheme method (Difco, Sparks, MD, USA) [39].

### 2.5. Antimicrobial Susceptibility Testing

The antimicrobial susceptibility profile for *Salmonella* isolates (*n* = 75) was investigated against 8 different antimicrobial agents using the Kirby–Bauer disc diffusion method (Oxoid Ltd., Basingstoke, UK). The sensitivity pattern was interpreted according to the guidelines of Clinical and Laboratory Standards Institute [40]. The antibiotics selected were those commonly used in the poultry industry, including Cefotaxime (CTX, 30 μg), Ceftriaxone (CRO, 30 μg), Ampicillin–Sulbactam (SAM, 10 μg), Amoxicillin–clavulanic (AMC, 30 μg), Impenem (IPM, 10 μg), Sulfamethoxazole–trimethoprim (SXT, 25 μg), Ciprofloxacin (CIP, 5 μg) and (Gentamicin CN, 10 μg) (Oxoid Ltd., Basingstoke, UK). The diameter of the inhibition zone was measured compared with the zone size interpretation chart and was graded as susceptible (S), intermediate (I), and resistant (R).

### 2.6. Molecular Detection of Salmonella-Related Biofilm Gene

Nucleic acid specific for each *Salmonella* serotypes was separately extracted using the QIAamp DNA Mini kit (Qiagen, Germany, GmbH). Briefly, 200 µL of the sample suspension was added to a volume of 10 µL proteinase K, and 200 µL lysis buffers were then incubated at 56 °C for 10 min. Then, 200 µL of ethanol (100.0%) was added to the lysate. Then, the lysate was rinsed and centrifuged. Lastly, the nucleic acid of each sample was eluted with 100 µL of elution buffer provided with the kit. Additionally, the quality of the extracted DNA from each sample was verified spectrophotometrically by means of a NanoDrop device and agarose gel electrophoresis.

For operating the conventional type PCR, the reactions were used in a volume of 25 µL, including a 12.5 µL Emerald Amp Max PCR Master Mix (Takara, Maebashi, Japan), 1 µL of the targeted primer (20 pmol concentration), 4.5 µL of Rnase free-water and 6 µL of sample DNA. The reaction was achieved in an applied bio-system 2720 thermal cycler. The amplification of the *csgd* gene specific to *Salmonella* serotypes is shown in Table 1 [41]. The parameter of thermocycling started with an initial denaturation cycle at 94 °C for 5 min followed by 30 cycles at 94 °C for 30 s, and the annealing at 50 °C for 40 s. The finishing extension stage was at 72 °C for 7 min. The PCR products were separated on a 1.0% agarose gel (Applichem, Darmstadt, Germany, GmbH) in a 1 × Tris/Borate/EDTA (TBE) buffer at gradients of 5 V/cm. For gel evaluation, 40 µL of the PCR products was inserted into each gel hole and the gel was interpreted by a gel documentation device (Alpha Innotech, Biometra, Göttingen, Germany), and the data point was assessed by the UVP 97-0244-01 DigiDoc-IT Imaging computer software System.

### 2.7. Synthesis of MOF

Three MOF composites Cu-MOF (MOF1), Cu/Ni-MOF (MOF2) and Cu/Ni/ CO-MOF (MOF3) were made using a solvothermal technique. For Cu-MOF (MOF1), the metal precursor Cu (NO_3_)_2_·4H_2_O (3 mmol) was injected together with 3 mmol of 2-amino terephthalic acid, which was dissolved in 120 mL of N, N-dimethylformamide (DMF). A magnetic stirrer was used to agitate the mixture until it was homogeneous. The solution was treated in a Teflon-lined stainless steel autoclave. It was progressively heated to 160 °C for 12 h before being cooled to room temperature. The combination underwent filtering, centrifuging, and a 24 h 60 °C oven drying process.

The same preparation was performed for Cu/Ni-MOF (MOF2) and Cu/Ni/Co-MOF (MOF3) followed by a Cu/MOF (MOF1) procedure with different molar ratios.

### 2.8. Characterization of MOF

The crystal structures of MOF composites were investigated using X-ray diffractometer (Cu-Ka radiation) at a voltage of 40 kV and a current of 35 mA (Malvern Panalytical Ltd, Malvern, United Kingdom). The FTIR spectroscopic spectra of MOF composites was examined using a Bruker Vertex 70 (Thermo ESCALAB 250XI, Waltham, MA, USA). Germany’s Zeiss Sigam 500 VP Analytical FE-SEM (Carl Zeiss, Jena, Germany) performed the morphology investigation using a scanning electron microscope.

### 2.9. Antimicrobial Assessment of MOF

#### 2.9.1. Inculum Preparation

The antimicrobial efficacy of MOF was tested on the obtained 75 isolates of *Salmonella* that were resistant to one or more of the tested antibiotics using agar well diffusion methodology. Briefly, the isolates of *Salmonella* were grown overnight on nutrient agar plates. A separate colony from each isolate was picked, suspended in physiological saline (NaCl, 0.9%) and McFarland reaction was justified at 1.5 × 10^8^ CFU/mL.

#### 2.9.2. Well Agar Preparation

Tryptone soya agar (TSA) media were weighted, prepared and autoclaved at 121 °C for 15 min, then allowed for cooling to 55 °C. The previous prepared bacterial suspensions were added at volumes of 1 mL of bacterial suspension/1 L of the cold media, followed by the inoculated agar, and it was allowed to solidify. Lastly, approximately 40–45 µL of the freshly prepared 0.01 and 0.05 mg/mL concentrations of MOF were individually loaded in the agar wells 6 mm in diameter, and the inoculated plates were aerobically incubated at 37 °C for 24–48 h. After incubation, the growth inhibition zone diameters were measured and evaluated.

### 2.10. Statistical Analysis

Data obtained were recorded and the frequency of *Salmonella* in the collected samples, and the antimicrobial efficacy of tested antibiotic and MOF were calculated using non-parametric tests (Chi-Square Test) using SPSS (Inc. version 22.0, Chicago, IL, USA).

## 3. Results and Discussion

*Salmonella* spp. specimens were frequently found in 75 (25.0%) of 350 samples collected from poultry shops that were under investigation (*p* < 0.01) (Table 2); they were most frequently recovered from cutting boards (27.5%), followed by rinse water and shop employee hands (22.5 and 22.0%, respectively). The findings of the current study are similar to those reported by Ahmed et al. [43], who obtained *Salmonella* by rate of 18.7 and 16.9%., respectively. Meanwhile, our findings were unlike those found by Orabi et al. [44], who detected *Salmonellae* in a rate of 10.0%. Our observations propose the prospect that the environment has a potential role in circulating the pathogen between animals and human populations. This might be attributed to the lack of hygiene and the misuse of antibiotics in the treatments of human or animal diseases [45]. Serotypes of *Salmonella* spp. that were recognized in the current study included *S. typhimurium*, *S. infantis*, *S. virchow*, *S. paratyphi C*, *S. heidelberg*, and *S. derby*.

Remarkably, reports similar to out findings denoted by Ahmed et al. [43], who corroborated that the surface swabs, showed the highest isolation rates (30.0%) compared to those of the hand swabs (15.0%). Thiruppathi et al. [46] also found *Salmonella* in a rate of 18.75% in the cutting board, higher than that recorded in the hand swab (14.29%). The recovery of *Salmonella* from environmental samples was most frequently reported in cutting boards, followed by the rinsing water, worker hands, intestinal swabs, de-feathering machines, and cutting knives at the rate of 27.5, 22.5, 22.0, 20.0, 15.0%, respectively. Nearly similar observations were reported by Sharada et al. [47]. The prevalence of these serotypes in the obtained samples highlights the potential health hazards to shop workers and subsequently poultry consumers through the food chain (CDC, 2009). Additionally, our observations indicated the role of the cutting boards in spreading various *Salmonella* serotypes that were similar to those denoted by Akl et al. [48], who reported that the cutting boards played a potential role in the cross-contamination of *S*. *typhimurium*, *E. coli* 0157: H7, and *Listeria monocytogenes*, and suggested that the highest incidence of *Salmonellae* from cutting boards indicate the lack of hygiene in the cleaning of these surfaces that enable the survival and multiplying different pathogens. Furthermore, it is thought that even with daily washing, the rough and porous surface of the chopping boards makes them more difficult to clean and sanitize, allowing the survival of different *Salmonella* strains for a prolonged period outside the living host in a sessile form furthermore. These studies also showed that *Salmonella* can establish itself in the environment for at least 24 h after becoming free from fresh food sources [49,50].

**Figure 1:**
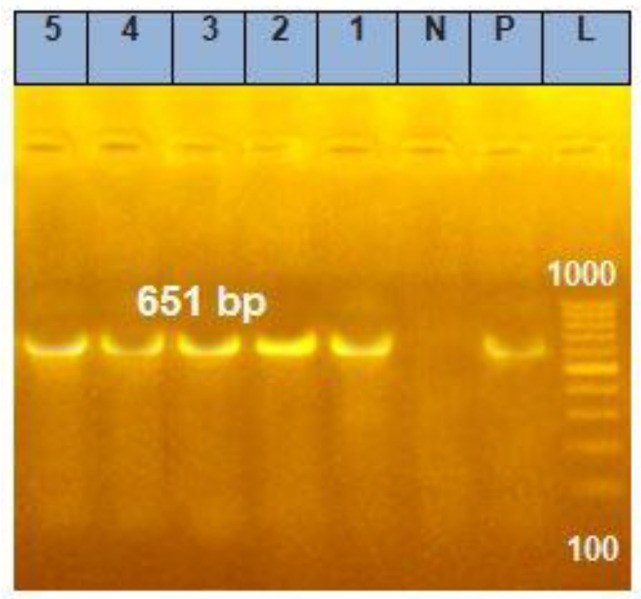
Agarose gel electrophoresis for the identified *Salmonella* PCR products with the biofilm gene marker (*Csgd*), amplified 651 base pair (bp). Lane (L); 100 bp Ladder marker, Lane (1–5); the examined isolates, Lane Pos; Positive control, Lane Neg; Negative control.

Figure 1 refers to the *csgd*-biofilm-related gene. It was detected in all of the randomly selected *Salmonella* traits obtained from the different samples; this gene is responsible for the ability of the microbe to form a biofilm on the biotic surfaces and the ability to adhere to abiotic surfaces as well [51]. This characteristic enables the pathogen to surround itself with an extracellular polymeric matrix (phospholipids, proteins, polysaccharides, and nucleic acids) [52,53,54]. With regard to the in vitro sensitivity of the antimicrobial profile of *Salmonella* against the tested antibiotics (Table 3), all examined traits showed multi-drug re-resistance to most of the tested antibiotics (*p* < 0.01), and the examined traits were extremely resistant to amoxicillin–clavulanic acid with different degrees (*p* < 0.01), mainly for the isolates collected from the intestinal swab and cutting board samples (100.0%) followed by those examined from the hand swabs, cutting knives, and rinsing water at rates of 90.1, 88.9, and 88.8%, respectively. Furthermore, isolates obtained from cutting knives were considerably resistant to gentamycin (100.0%), followed by those obtained from intestinal swabs, hand swabs, and cutting boards at 93.3, 90.1%, and 8.9%, respectively. Similar findings were investigated by Doyle et al. [55], Zishiri et al. [56], Nabil and Younis [57] and Orabi et al. [44]. Notably, the exuding rates of microbial resistance to most of the investigated antibiotics in this study were not surprising, which suggests the abuse and/or overuse of most common therapeutic antimicrobial classes utilized in the field of the poultry industry with risk of transmission of this resistance to human traits through the food chain [58,59].

From the data presented in Table 4, it is clear that Cu/MOF showed different degrees of sensitivity to diminish the growth of *Salmonella* isolated from animals, humans, and the environment, which generally increased with an increase in its concentration at p-value < 0.01. Furthermore, our finding of in vitro sensitivity of antimicrobial of Cu/Ni-MOF and Cu/Ni/Co-MOF showed promising results at both concentrations, 0.01 and 0.05 μg/mL, respectively, against different isolates of *Salmonella* obtained either from poultry, human or the environment. MOFs are a group of compounds that involve inorganic and organic secondary building units (SBUs) attached to a three-dimensional extended frame with capacity holes. MOFs receive a lot of attention in various applications [60]. Additionally, the microporous architectures and ultra-high surface area of MOFs make them appealing for a variety of applications, such as delivery systems for antimicrobial drugs due to the antibacterial properties of MOF constituents (either metal ions or ligands). Additionally, the MOF long-term regulated release rate boosts their importance as nano-carriers for the delivery of pharmaceuticals [61].

**Figure 2:**
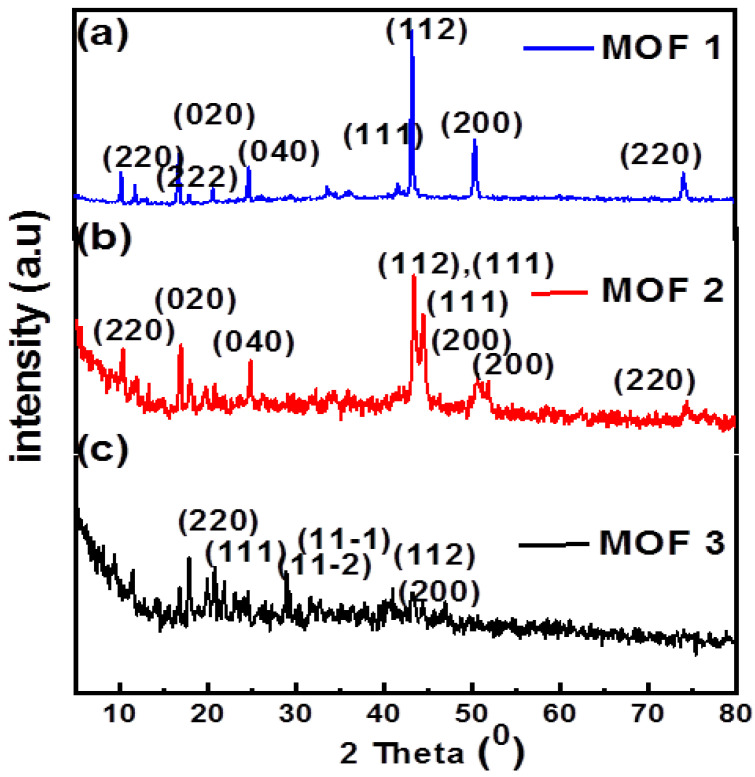
XRD patterns of (**a**) Cu-MOF (MOF 1), (**b**) Cu/Ni-MOF (MOF 2) and (**c**) Cu/Ni/Co-MOF (MOF 3).

Concerning the incontrovertible bactericidal effect of Cu-MOF, Cu/Ni-MOF, and Cu/Ni/Co-MOF in control of *Salmonella* spp., it could be denoted by X-ray diffraction, which is primarily used to identify potential metal complex structures and additional structural evidence [62]. In Figure 2a for Cu-MOF (MOF 1), most of the peaks are consistent with the literature, which is matched with cards 89-2838 and 04-0836 for copper nanoparticles [63,64], and carbon is matched with JCPDS card no. 732058 [65,66]. These results support the generation of pure Cu nanoparticles with cubic face-centered shapes. Peak values for the Cu-MOFs (MOF1), 2θ at 17.30 and 26.80°, are related to planes at (020) and (040), respectively. Numerous other peaks are observed between 2 at 10° and 50°. The peaks showed at 10.2, 11.9, 13.01, 16.8, 18°, 20.7°, and 24.7° that correspond to (110), (220), (020), (333), (211), (202), and (222) lattice planes. This is effective in producing crystals with a very high level of crystallinity and intensity [67,68]. For Cu/Ni-MOF (MOF 2) in Figure 2b, Ni and Cu have a polycrystalline structure, as shown by the XRD peaks of the Ni/Cu MOF at 45.2°, 44.7°, 50.4°, and 51.9°, which contain the (111) and (200) planes, respectively. This supports the interaction of bimetal centers [69]. As shown in Figure 2c, the patterns of the Co-MOFs are attributed to the clear and distinct diffraction peaks at 2θ = 9.2°, 11.2°, 14.3°, 16.8°, 18.6°, 21.4°, 22.6°, 23.9°, 28.9°, 30.5°, 32.7°, 33.5°, and 39.8°, suggesting good crystallinity of the as-prepared Materials [70,71].

**Figure 3:**
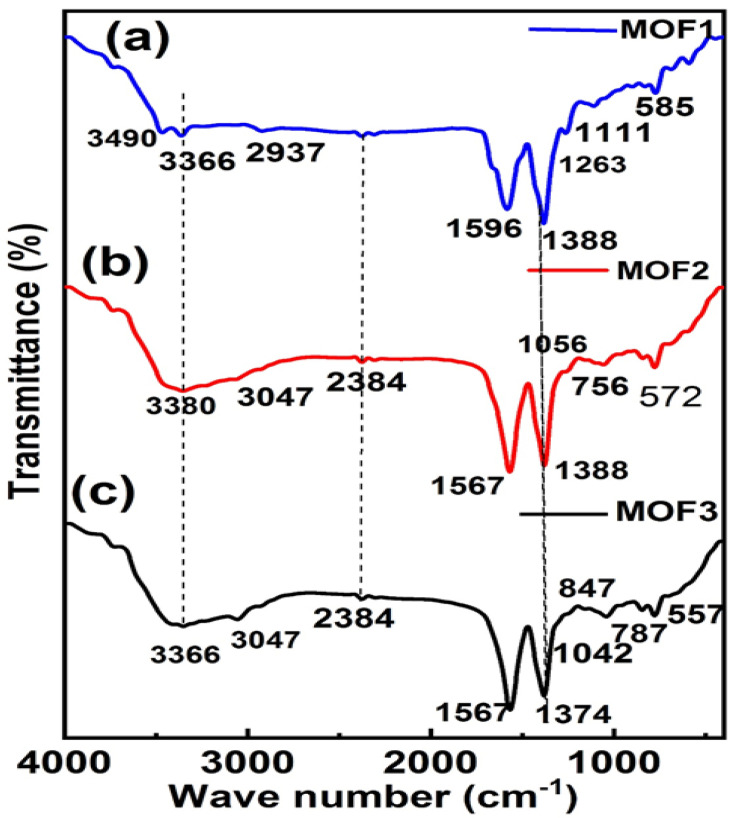
FT-IR spectra of (**a**) Cu-MOF (MOF 1), (**b**) Cu/Ni-MOF (MOF 2) and (**c**) Cu/Ni/Co-MOF (MOF 3).

In the FT-IR spectrum of Cu-MOF (MOF 1) in Figure 3a, bands are observed at 3490 cm^−1^ and 3366 cm^−1^, corresponding to O-H stretching and N-H stretching, respectively. The coordination link between the organic ligand and metal may be responsible for the lower frequencies of both bands [63]. Similar to alkene C=C str, N-H bending and O-H stretching frequencies are recorded at 1596 cm^−1^, 1388 cm^−1^, and 1111 cm^−1^, respectively. An aromatic amine C-N stretching peak is observed at 1263 cm ^− 1^. Additionally, an effective absorption band at 585 cm^−1^ in the fingerprint region is linked to the vibrations of the Cu-O functional group [72]. The bands at 3440 cm^−1^ and 3108 cm^−1^, which are attributed to the stretching vibration of water molecules, provide evidence of coordinated H_2_O molecules within the Cu/Ni-MOF (MOF 2) structure, as shown in Figure 3b. Figure 3b shows that the asymmetric and symmetric H-N group vibration bands fill the space between 3300 and 3000 cm^−1^ [73]. While the apparent peak at 587 cm^−1^ demonstrates the presence of Ni and its coordination with the COOH group oxygen, the C-H peak appears at 754 cm^−1^ [74]. The gap between these two bands indicates the interaction of the COO^−1^ group of the linker with Ni metal via the bidentate mode of linking. Symmetric and asymmetric stretching vibrations of the COO^−1^ group produce reasonable adsorption bands at 1567 and 1388 cm^−1^, respectively [75]. The metal–oxygen–hydrogen bending vibration (Ni-OH, Co-OH, or Ni-Co-OH) is responsible for the absorption at approximately 557 cm^−1^ in this framework [76].

**Figure 4:**
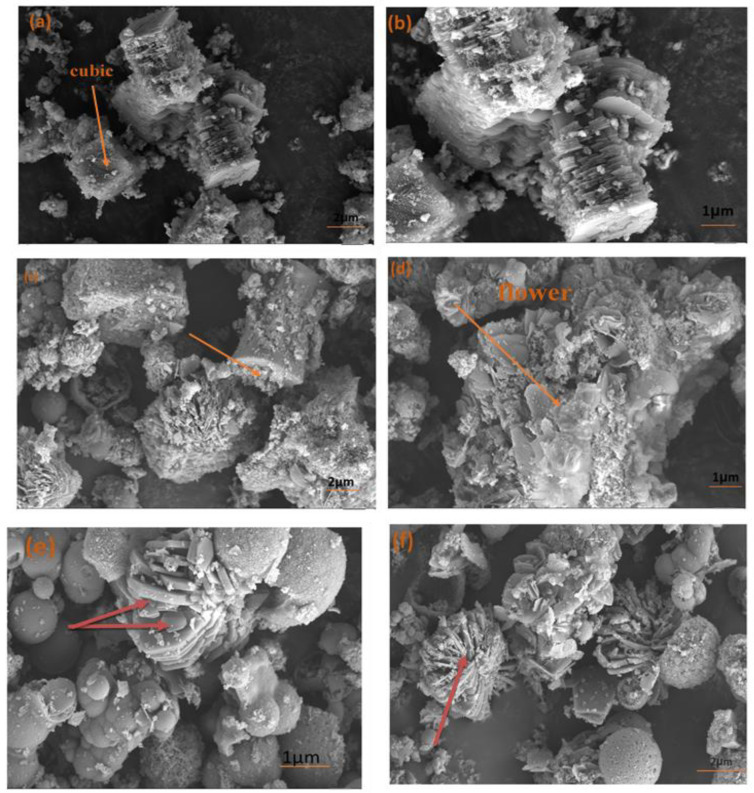
SEM images of Cu-MOF (MOF 1) (**a**,**b**), Cu/Ni-MOF (MOF 2) (**c**,**d**), Cu/Ni/Co-MOF (MOF 3) (**e**,**f**).

Figure 4 illustrates the use of field emission scanning electron microscopy (FE-SEM) to describe the morphology of Cu-MOF (MOF1), Cu/Ni-MOF (MOF 2), and Cu/Ni/Co-MOF (MOF 3). The Cu-MOF particles in Figure 4a,b for (MOF 1) demonstrate the existence of fully developed cubic microcrystals [77]. The microscopic flakes of Cu/Ni-MOF (MOF 2), which are around ten nanometers in size, have a flower-like morphology as shown in Figure 4c,d [75]. Figure 4e,f demonstrates that it is a conventional cuboid shape with a rough, porous surface, a flower shape, and a spherical shape [78].

## 4. Conclusions

This study revealed the important role of the environment in the circulation of pathogens (*Salmonella*) between poultry and human consumers. Moreover, this study clarified the ability of the environment to act as a source of antimicrobial resistance genes, and the role of microbial biofilm formation in this resistance. Furthermore, our study demonstrated the promising effects of MOF in controlling *Salmonella* spp. We suggest that further research should be conducted to investigate the efficacy of MOF against different disinfectants used to control *Salmonella* spp. in the environment and the biocidal and biotoxicity of MOFs in mammalian cell lines.

## Figures and Tables

**Table 1:** Gene sequences of the targeted *csgd*-biofilm gene marker specific for *Salmonella* in the current study.

Target Gene	Primers Sequences	Amplified Segment (bp)	References
*csgd*	TTACCGCCTGAGATTATCGT	651	[42]
	ATGTTTAATGAAGTCCATAG		

**Table 2:** Frequent distribution of isolated *Salmonella* serotypes in the examined samples.

Samples/Swabs	No. Examined	No. Positive	Percentage (%)
Intestinal	140	30	21.4
Hand	50	11	22.0
Surface (Cutting broad)	40	11	27.5
Cutting knives	40	6	15.0
Rinsing water	40	9	22.5
Defeathering machine	40	8	20.0
Total	350	75	25.0

X_2_ = 84.00 *p*-value < 0.01.

**Table 3:** In vitro antimicrobial profile of tested antibiotics against *Salmonella* serotypes obtained from the examined samples.

	**Antibiotic Sensitivity**	**Positive Samples** **(No.)**	**Antibiotics Used**																				
**Samples/Swabs**																							
-			**Cefotaxime ** **(CTX, 30 μg)**			**Ceftriaxone ** **(CRO, 30 μg)**			**Ampicillin-Sulbactam (SAM, 10 μg)**			**Amoxicillin-Clavulanic (AMC, 30 μg)**			**Sulfamethoxazole-Trimethoprim ** **(SXT, 25 μg)**			**Gentamicin ** **(CN, 10 μg)**			**Ciprofloxacin ** **(CIP, 5 μg)**		
-			**S**	**I**	**R**	**S**	**I**	**R**	**S**	**I**	**R**	**S**	**I**	**R**	**S**	**I**	**R**	**S**	**I**	**R**	**S**	**I**	**R**
Intestinal swab		30	9 30.0	4 13.3	17 56.7	10 33.3	- 0.0	20 66.7	8 26.7	1 3.3	21 70.0	- 0.0	- 0.0	30 100.0	4 13.3	2 6.7	24 80.0	- 0.0	2 6.7	28 93.3	2 6.7	4 13.3	24 80.0
Hand swab		11	1 9.1	3 27.3	7 63.6	4 36.4	2 18.2	5 72.7	2 18.2	1 9.1	8 72.7	1 9.1	- 0.0	10 90.1	- 0.0	2 18.2	9 81.8	- 0.0	1 9.1	10 90.1	- 0.0	1 9.1	10 90.1
Cutting broad Swab		9	1 11.1	1 11.1	7 63.6	2 22.2	1 11.1	6 66.7	- 0.0	1 11.1	8 88.9	- 0.0	1 11.1	8 88.9	2 22.2	- 0.0	7 63.6	1 11.1	- 0.0	8 88.9	2 22.2	1 11.1	6 66.7
Cutting knives swab		6	2 33.3	- 0.0	4 66.7	1 16.7	1 16.7	4 66.7	2 33.3	1 16.7	3 50.0	- 0.0	- 0.0	6 100.0	2 33.3	- 0.0	4 66.7	- 0.0	- 0.0	6 100.0	2 33.3	- 0.0	4 66.7
Rinsing water		11	2 18.2	- 0.0	9 81.8	1 9.1	3 27.3	7 63.6	2 18.2	- 0.0	9 81.8	1 9.1	1 9.1	9 81.8	3 27.3	- 0.0	8 72.7	2 18.2	- 0.0	9 81.8	3 27.3	- 0.0	8 72.7
Defeathering machine		8	2 25.0	1 12.5	5 62.5	- 0.0	1 12.5	7 87.5	2 25.0	1 12.5	5 62.5	2 25.0	- 0.0	6 75.5	1 12.0	- 0.0	7 87.5	2 25.0	1 12.5	5 62.5	- 0.0	1 12.5	7 87.5

*p*-value < 0.01.

**Table 4:** In vitro sensitivity of Cu/MOF, Cu/Ni/MOF, and Co/Cu/Ni/MOF against obtained *Salmonella* serotypes from the examined samples.

Samples	Positive Samples (No.)	Cu/MOF				Cu/Ni/MOF				Co/Cu/Ni/MOF				*p*-Value
		0.01		0.05		0.01		0.05		0.01		0.05		
		R	S	R	S	R	S	R	S	R	S	R	S	
Intestinal swab	30	12 40.0	18 60.0	10 33.3	20 66.7	14 46.7	16 53.3	13 43.3	17 56.6	8 26.7	22 73.3	6 20.0	24 80.0	0.000
Hand swab	11	4 76.7	7 23.3	3 27.3	8 72.7	5 45.4	6 54.5	4 36.4	7 63.6	3 27.3	8 72.7	2 18.2	9 81.2	0.000
Cutting broad Swab	9	7 77.7	2 22.2	5 55.5	4 44.4	4 44.4	5 55.6	3 33.3	6 66.7	1 11.1	8 72.7	2 18.2	7 77.8	0.004
Cutting knives swab	6	6 66.7	3 33.3	4 44.4	5 55.6	3 50.0	3 50.0	3 50.0	3 50.0	2 33.3	4 66.7	2 33.3	4 66.7	0.002
Rinsing water	11	5 45.4	6 64.5	4 36.3	7 63.6	5 45.4	6 54.5	4 36.4	7 63.6	3 27.3	8 72.7	3 27.3	8 72.7	0.014
Defeathering machine	8	5 62.5	3 37.5	4 50.0	4 50.0	4 50.0	4 50.0	2 25.0	6 75.0	1 12.5	7 87.5	1 12.5	7 87.5	0.003

*p*-value = 0.000.

## Abbreviations

MOFs	Metal Organic Frameworks
MOF1	Cu-MOF
MOF2	Cu/Ni-MOF
MOF3	Cu/Ni/Co-MOF
SEM	Scan Electron Microscope
XRD	X-Ray Diffraction
FTIR	Fourier-Transform Infrared Spectroscopy
R	Resistance
I	Intermediate
S	Susceptible
